# Laterality across languages: Results from a global dichotic listening study using a smartphone application

**DOI:** 10.1080/1357650X.2014.997245

**Published:** 2015-01-14

**Authors:** Josef J. Bless, René Westerhausen, Janne von Koss Torkildsen, Magne Gudmundsen, Kristiina Kompus, Kenneth Hugdahl

**Affiliations:** ^a^ Department of Biological and Medical Psychology, University of Bergen, Bergen, Norway; ^b^ NORMENT Center of Excellence, University of Oslo, Oslo, Norway; ^c^ Department of Special Needs Education, Faculty of Educational Sciences, University of Oslo, Oslo, Norway; ^d^ Division of Psychiatry, Haukeland University Hospital, Bergen, Norway; ^e^ Department of Radiology, Haukeland University Hospital, Bergen, Norway

**Keywords:** Language lateralization, Dichotic listening, Laterality, Smartphone application, Field experiment

## Abstract

Left-hemispheric language dominance has been suggested by observations in patients with brain damages as early as the 19th century, and has since been confirmed by modern behavioural and brain imaging techniques. Nevertheless, most of these studies have been conducted in small samples with predominantly Anglo-American background, thus limiting generalization and possible differences between cultural and linguistic backgrounds may be obscured. To overcome this limitation, we conducted a global dichotic listening experiment using a smartphone application for remote data collection. The results from over 4,000 participants with more than 60 different language backgrounds showed that left-hemispheric language dominance is indeed a general phenomenon. However, the degree of lateralization appears to be modulated by linguistic background. These results suggest that more emphasis should be placed on cultural/linguistic specificities of psychological phenomena and on the need to collect more diverse samples.

For the past 50 years, dichotic listening has served as a non-invasive method for the study of hemispheric lateralization of speech perception (Hugdahl, 2011). Its hallmark finding is a preference to report the right-ear over the left-ear stimulus of two, simultaneously presented consonant-vowel (CV) syllables or words, a phenomenon called the right-ear advantage (REA; Bryden, 1988; Hugdahl, 1995; Kimura, 1961). The REA is an indicator of left-lateralized processing of language and has been validated with a variety of methods, such as functional magnetic resonance imaging (fMRI; e.g., Hund-Georgiadis, Lex, Friederici, & von Cramon, 2002; van den Noort, Specht, Rimol, Ersland, & Hugdahl, 2008; Westerhausen, Kompus, & Hugdahl, 2014), positron emission tomography (Hugdahl et al., 1999), the Wada procedure (e.g., Hugdahl, Carlsson, Uvebrant, & Lundervold, 1997; Strauss, Gaddes, & Wada, 1987), as well as lesion studies (e.g., Gramstad, Engelsen, & Hugdahl, 2003; Pollmann, Maertens, von Cramon, Lepsien, & Hugdahl, 2002; Sparks, Goodglass, & Nickel, 1970).

Although the REA is one of the best-described perceptual phenomena in neuropsychological research, it is based on results obtained from small samples in the laboratory. In addition, subjects typically fall into the so-called “WEIRD”-category, that is, they come from Western, educated, industrialized, rich and democratic cultures (Jones, 2010). Moreover, Arnett (2008) reported on the dominance of samples from English-speaking countries, and particularly the USA, as they are represented in 82% of studies published in *APA* journals. One main reason for this state of affairs in psychological research is the constraints that are accompanying running experiments in the laboratory, which make it impossible to collect large-scale data from different countries and cultures within the same experimental design or paradigm and within reasonable time. As a consequence, empirical evidence for models and theories of general psychological phenomena is heavily biased towards a minority, about 5% of the world's population (see Arnett, 2008), and any findings made on the basis of this minority may not necessarily generalize to all humans (see Henrich, Heine, & Norenzayan, 2010). Indeed, previous studies have shown that cultural differences exist for complex tasks, e.g., in fairness and economic decision-making (Henrich et al., 2006), as well as for basic perceptual phenomena such as in the Müller-Lyer illusion (Segall, Campbell, & Herskovits, 1966). Thus, universality should not be assumed before the phenomenon in question has been explored across a range of cultures. The internet and smartphones offer an opportunity for a paradigm shift in psychological research by allowing to collect more diverse and larger samples, under various real-life conditions, yet with the same experimental paradigm (Gosling, Sandy, John, & Potter, 2010; see Miller, 2012). In this way, data collected globally should provide results that better represent the world's population and at the same time identify cultural/linguistic specificities among sub-populations.

In this paper we present the results of a large-scale (>4000 subjects) and international (>60 native languages) field experiment on language lateralization using a mobile-app for data collection (*iDichotic*). The app served as a self-administered dichotic listening test based on the CV-paradigm (Hugdahl & Andersson, 1986), and it has previously been shown to produce both reliable and valid results (Bless et al., 2013). App-users from around the world could participate in this field-experiment by submitting their test results to our database. This is the largest and (culturally/linguistically) most diverse sample of subjects that has been tested for language lateralization, allowing us to explore if the phenomenon in question (i.e., the REA, indicating left-lateralized processing of language) is indeed a universal phenomenon (generality of the REA). Also, having data collected from individuals of different language backgrounds with exactly the same experimental paradigm, allowed us to test for differences between language groups (specificity of the REA). We expected to find the REA irrespective of language background (generality) and sought to examine the differences in the magnitude of the REA between languages (specificity). Based on our previous study in a smaller sample using the same paradigm (Bless et al., 2013), we would expect to find differences in the REA between language groups. However, other studies using different dichotic listening paradigms (multiple responses per trial) have not found cross-cultural differences (Cohen, Levy, & McShane, 1989; Nachshon, 1986). In addition, we also expected the size of the REA to be modulated by sex (males > females), handedness (right-handers > left-handers) and age (increasing REA with age), as reported in previous studies (Hirnstein, Westerhausen, Korsnes, & Hugdahl, 2013; Hiscock, Inch, Jacek, Hiscock-Kalil, & Kalil, 1994; Hugdahl, 2003).

## METHODS

### Database and samples

**Figure 1.**
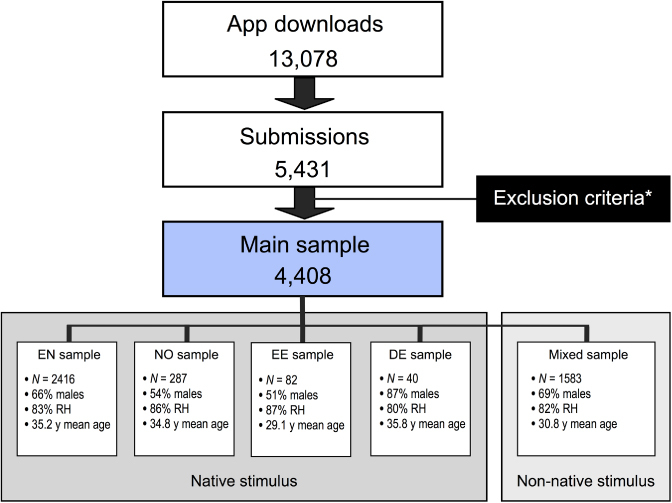
Flow-chart depicting sample selection process and characteristics of sub-samples. *See separate section under Methods. RH = Right-handed. y = years. N = number of subjects. EN = English; NO = Norwegian; EE = Estonian; DE = German.

The complete database consists of 5,431 submissions from *iDichotic* app-users around the world. The app was promoted via various media channels (radio, web, newspapers). Since the data were collected under uncontrolled experimental conditions, through self-administration, a number of exclusion criteria were applied (see below) to control for this. This resulted in the exclusion of 1,022 data sets (19%) and a remaining main sample size of 4,408 participants (66% males, 83% right-handers, mean age 33.6) from 64 different native language backgrounds for the main analysis. Two thousand eight hundred twenty-five participants received the stimulus materials in their native language, while 1,583 participants received the materials in a non-native language (see Figure 1 for an overview). The experiment was carried out in accordance with the Declaration of Helsinki and informed consent was obtained prior to submission of the test results.

### Material

The app *iDichotic* was developed in-house using the iOS software development kit (Apple Inc., Cupertino, CA) and is available free of charge on the App Store, which makes it compatible with iPhone, iPad and iPod touch devices. The test is based on the standard Bergen dichotic listening paradigm (Hugdahl, 2003; Hugdahl & Andersson, 1986). In this paradigm, the stimuli consist of the six CV-syllables /ba/, /da/, /ga/, /ta/, /ka/ and /pa/, presented via headphones in pairs, one syllable played in the right-ear channel and the other syllable played simultaneously in the left-ear channel. In this way, all possible combinations are presented forming 30 unlike pairs (e.g., /ba/-/ka/, /ta/-/ka/ etc.) and 6 like pairs (e.g., /da/-/da/, /ta/-/ta/). The stimuli were available in four different language-sets, that is, the syllables were spoken with constant intonation and intensity by native speakers of (British) English, Norwegian, German and Estonian. The six stimuli of each language-set varied in their length due to differences in the voice-onset time (VOT) of the consonant. Two types can be distinguished: those with long VOT (i.e., /pa/, /ta/, /ka/) and those with short VOT (i.e., /ba/, /da/, /ga/) (see Rimol, Eichele, & Hugdahl, 2006). Previously, this stimulus set-up has yielded laterality estimates that were successfully validated against the sodium amytal procedure (e.g., Hugdahl et al., 1997; Strauss et al., 1987), as well as minimally invasive (e.g., Hugdahl et al., 1999) and non-invasive neuroimaging techniques (e.g., Van der Haegen, Westerhausen, Hugdahl, & Brysbaert, 2013; van den Noort et al., 2008; Westerhausen et al., 2014). Also, independent of length difference within the stimulus set of one language, there were differences in the overall length between the four language-sets (English: 480–550 msec, Norwegian: 400–500 msec, German: 320–380 msec, Estonian: 320–390 msec). This is due to the syllables being recorded to sound as natural/native as possible, with the intention to preserve the specific phonetic character of each language. The inter-stimulus interval was kept constant for all language-sets at 4,000 msec.

### Procedure

The experiment was implemented as a self-administration test. First, the *iDichotic* app had to be downloaded from the App Store and installed on a mobile device (e.g., iPhone). As part of the app and before the start of the test, participants were asked to report on a set of variables, including stimulus language (English, Norwegian, German, Estonian), age (in years), sex, handedness (3 alternatives: right, left, both) and native language (including the main English dialects). They also performed a simple hearing test to control for hearing asymmetries that would bias the test results (see exclusion criteria below). In this test, using a horizontal volume scroll-bar, a 1000 Hz tone had to be adjusted to the point it became “just inaudible” (separate for left and right ear), Participants were reminded (via a pop-up window) to wear the headphones in correct orientation (left-channel headphone on the left ear, right-channel headphone on the right ear) and adjust the main volume to a comfortable level. In the next step, test instructions were presented on the screen corresponding to the non-forced condition of the Bergen dichotic listening paradigm (Hugdahl, 2003). That is, participants were instructed to listen to a series of syllable pairs (36 pairs) and respond after each trial (1 pair per trial) by selecting (on the touch-screen) the syllable he/she had heard best. Participants had 4 sec to respond before the next syllable pair was presented. A response was counted as correct when the selected syllable matched the syllable presented to either the right or left ear on each trial; if the response did not match either syllable, or no response was given, it was counted as an error. The error was calculated as follows:

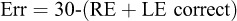

The laterality index (LI) was calculated to quantify the magnitude of language lateralization using this formula:

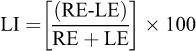

Thus, a REA (left-hemisphere language dominance) was indicated by a positive LI, while a left-ear advantage (right-hemispheric language dominance) was indicated by a negative LI. At completion of the 3-minute long test, the results were displayed including the option to submit the results (see below) to our database.

### Data collection

The data were collected via secure file transfer protocol during the period between 10 August 2012 and 27 January 2014. The majority of the data was collected during a six-week period as a result of a series of media promotions of the app. The end point of data collection was arbitrary. Since the aim was to attain the largest possible sample, no a priori sample size was calculated, but effect sizes were considered in the interpretation of effects. The submission of test results was optional, anonymous at all times and required accepting the terms of informed consent (via pop-up notification). The submitted file contained the test scores, participant-variables, submission date and app-ID (date of app download + random number).

### Exclusion criteria

First, in order to guarantee that a participant was able to identify the syllables we decided that at least 6 out of 30 (20%) total reports needed to be correct in dichotic trials. For homonym trials, 3 out of 6 (50%) were considered adequate. Further, cases that had an absolute LI of 100%, indicating that there was no correct identification of stimuli presented to one (the right or the left) ear were also excluded. Although it cannot be completely ruled out that this is in fact a “true” REA/LEA, data from laboratory experiments very rarely show a 100% ear advantage (see Hugdahl, 2003). Thus, we considered it more likely that most of these cases were artefactual, e.g., due to a unilateral hearing loss in one ear, or a broken headphone piece. Also, a hearing asymmetry of more than 20% was a criterion for exclusion. The threshold is deduced from previous experiments under soundproof conditions, which showed that an asymmetry of more than 6db (10% of normal conversation of 60db) would start affecting the size of the ear advantage (see Hugdahl, Westerhausen, Alho, Medvedev, & Hämäläinen, 2008). However, since we used a crude measurement of hearing ability, and given the potentially noisy environments in which it was conducted, we found it reasonable to double the threshold level. In addition, participants under the age of eight were excluded, since it cannot be expected that they were able to read and understand the instructions. From around the age of eight, however, performance approaches “adult-like” behaviour, as has been shown in previous longitudinal studies with the same paradigm (Westerhausen, Helland, Ofte, & Hugdahl, 2010). Finally, double submissions from the same participant were excluded, that is only the first results-submission was counted, in order to avoid re-test/practice effects.

### Data analysis

The data were analyzed with five analyses of variance (ANOVAs) using the right ear/left ear scores as dependent variable (for details see Appendix Table A1). The purpose of the first analysis (Analysis 1) was to explore our hypothesis on the generality of the REA irrespective of language background as well as the expected effects of sex (male, female), handedness (right-handers, left-handers, ambidextrous), age and the use of stimulus language (native, non-native syllables). Further, the role of “nativeness” was explored by investigating the effect of phonetic overlap on the ear scores (Analysis 2a/b). Phonetic overlap was defined as the degree in which the native language and the stimulus language overlap with regard to place of articulation of the six stop consonants. For example, a native English speaker using the English syllables is assigned an overlap of six, since native language and stimulus language have identical points of articulation (for details see Appendix Table A2). For this purpose, we looked at the two sub-samples separately, one with complete phonetic overlap (native-stimulus sample; Analysis 2a) and another with varying degrees of phonetic overlap (non-native stimulus sample; Analysis 2b). Finally, in order to examine the effect of language/dialect background independently of the stimulus language and compare language/dialect groups directly, we analyzed the language groups with more than 100 participants (English, Danish, Norwegian, Hindi, Chinese, Spanish; Analysis 3a), and the English language dialects separately (North American, British, Australian; Analysis 3b). Of note, non-right-handers (ambidextrous, left-handers) were excluded in Analysis 2a/b and Analysis 3a/b, since keeping the factor handedness would result in an ANOVA design with less than 10 subjects in one or more cells.

Across all analyses the effects of interest were: (1) the interaction of the factor Ear with the respective language group factor, indicating differences in the magnitude of the ear advantage, and (2) the main effects of the language group factor, representing differences in the overall (average across ears) performance level. In general, significant main and interaction effects were followed-up by pairwise *t*-test comparisons and lower level ANOVAs. In order to control the familywise error rate, the Bonferroni correction was applied in the omnibus ANOVAs (i.e., adjustment alpha = 0.05/5 = 0.01). The Fisher's least significant difference procedure (alpha = 0.05) was used for the post-hoc tests. In addition, effect-size measures were provided indicating the proportion of explained variance, i.e., 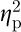. Mean LI scores were calculated for the various language/dialect sub-samples to describe the magnitude of the ear advantage in a commonly accepted form (formula see above). The statistical analyses were performed in PASW 18.0 (IBM SPSS, New York, USA).

## RESULTS

**Figure 2.**
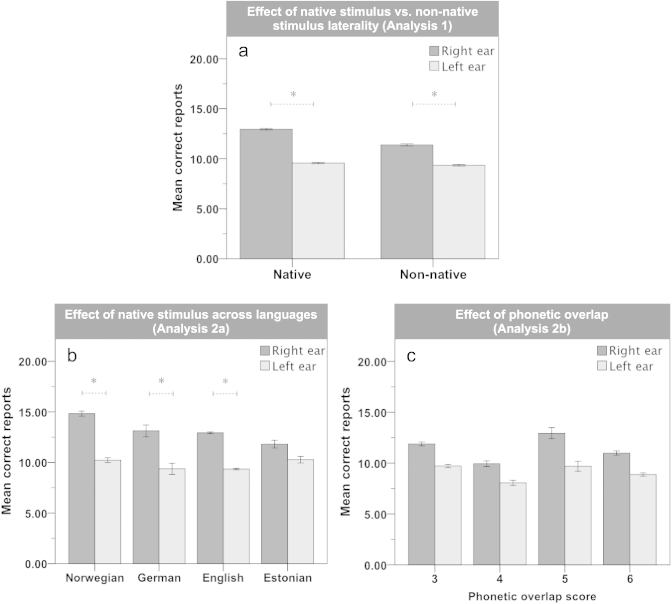
Three charts depicting the mean correct reports from the right and the left ear comparing (a) the native-stimulus vs. non-native stimulus sample, (b) native-stimulus sub-samples and (c) the non-native languages grouped by phonetic overlap. *y*-axis = 30 max. Error bars = standard error (SE). *Significant (*p* < .05) post-hoc pairwise comparisons.

In the main sample (Analysis 1), there was a significant main effect of *Ear, F*(1, 4395) = 30.78, *p* < .0001, 
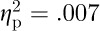
, right ear > left ear, equivalent to a REA of LI = 12.5 (*SE* = 0.4), and significant interactions between *Ear* × *Handedness*, *F*(2, 4395) = 10.29, *p* < .0001, 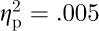, and *Ear* × *Stimulus language*, *F*(1, 4395) = 9.04, *p* < .01, 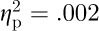, (see Figure 2a). Post-hoc analyses revealed that all three handedness groups showed a significant REA (*p* < .001). However, the interaction was based on right-handers displaying a significantly larger REA than left-handers (*p* < .0001), whereas there were no significant differences of the REA between the ambidextrous groups and either right- or left-handers (*p* > .05). With regard to stimulus language, both the native and non-native stimulus sub-samples showed a significant REA (*p* < .0001), with the native-stimulus sample displaying a significantly larger REA than the non-native sample [LI = 14.4 (*SE* = 0.5) and LI = 9.1 (*SE* = 0.7), respectively]. Furthermore, there were main effects of *Stimulus language, F*(1, 4395) = 47.70, *p* < .001, 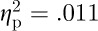, native > non-native, and *Age, F*(2, 4395) = 57.57, *p* < .0001, 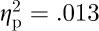. No other main or interaction effects were significant (*p*s > .043).

In the native-stimulus sample (Analysis 2a), there was a significant main effect of *Ear, F*(1, 2339) = 41.84, *p* < .0001, 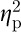
= .018, right ear > left ear, indicating a REA of LI = 15.4 (*SE* = 0.6), and a significant interaction between *Ear* × *Native language, F*(3, 2339) = 4.42, *p* < .01, 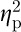
= .006, (see Figure 2b). Post-hoc analyses on the interaction effect showed a significant REA (*p* < .05) in all but the Estonian sub-sample, and the REAs differed significantly between certain native language groups (*p* < .05): Norwegian > (English > Estonian), but not between the other groups (*p*s > .05). Furthermore, there were main effects of *Native language, F*(3, 2339) = 35.02, *p* < .0001, η^2^ = .043, Norwegian > (English = German = Estonian), and *Age, F*(1, 2339) = 34.13, *p* < .0001, 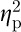
= .014. No other main or interaction effects were significant (*p*s > .36).

In the non-native stimulus sample (Analysis 2b), there was a significant interaction between *Ear* × *Sex, F*(1, 933) = 7.18, *p* < .01, 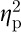 = .008. Post-hoc analyses of the interaction effect showed a significant REA in males (*p* < .0001) but not in females (*p* > .05), and the REA was significantly larger in males than females. Furthermore, there was a main effect of *Phonetic overlap, F*(3, 933) = 18.11, *p* < .0001, 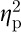 = .055, 3 > 4, 3 = 5, 3 > 6, 4 < 5, 4 < 6, 5 > 6. Importantly, the interaction of *Ear* × *Phonetic overlap* did not reach significance, *F*(3, 933) = 0.71, *p* < .54, (see Figure 2c). No other main or interaction effects were significant (*p*s > .01).

**Figure 3.**
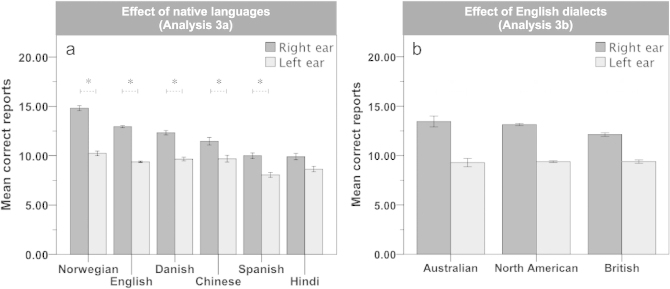
Charts depicting the mean correct reports from the right and the left ear comparing (a) the largest (*n* > 100) language groups of the main sample, and (b) the three largest (*n* > 30) English-dialect groups. *y*-axis = 30 max. Error bars = standard error (SE). *Significant (*p* < .05) post-hoc pairwise comparisons.

When looking at only the largest language groups (*N* > 100) of the main sample (Analysis 3a), there was a significant main effect of *Ear, F*(1, 3015) = 42.97, *p* < .0001, 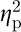 = .014, right ear > left ear, indicating a REA of LI = 14.1 (*SE* = 0.5), and a significant interaction between *Ear* × *Native language, F*(5, 3015) = 7.23, *p* < .0001, 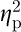 = .012, (see Figure 3a). Post-hoc analyses revealed that all of the language groups except for the Hindi group showed a significant REA (*p* < .05; for mean LI see Table A3). The interaction was based on the REA being larger in the Norwegian and the English group compared to all other groups (*p* < .05). Further, the REA was larger in the Norwegian than the English group (*p* < .05), while no significant differences were found between the other language-groups (*p*s > .13). Furthermore, there were main effects of *Native language, F*(5, 3015) = 63.59, *p* < .0001, 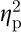 = .095, Norwegian > English > (Danish = Chinese) > Hindi = Spanish, and *Age, F*(1, 3015) = 33.97, *p* < .0001, 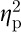 = .011. No other main or interaction effects were significant (*p*s > .01).

In the native English sample (Analysis 3b), there was a significant main effect of *Ear, F*(1, 2015) = 54.73, *p* < .0001, 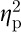 = .026, right ear > left ear, indicating a REA of LI = 15.2 (*SE* = 0.6). The effect of interest, i.e., the *Ear* × *Dialect* interaction was not significant, *F*(2, 2015) = 3.81, *p* = .02, 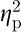 = .004, (see Figure 3b). Furthermore, there were main effects of *Dialect, F*(2, 2015) = 10.45, *p* < .0001, 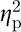 = .010, (North American = Australian) > British, and *Age, F*(1, 2015) = 31.77, *p* < .0001, 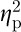 = .016. No other main or interaction effects were significant (*p*s > .14).

## DISCUSSION

The present study assessed the generality and specificity of the REA in a large, international sample, using a mobile-app version of the Bergen dichotic listening paradigm. Averaged across all sub-samples, an overall REA (LI = 12.5%) was found, and a significant REA emerged in most of the language sub-samples (see Appendix Table A3), indicating that left-lateralized processing of language indeed is a “universal” phenomenon. However, we also found specificity of the REA emerging as differences in magnitude between the languages groups. These differences appeared to cut across language families. For example, Danish showed a significantly smaller LI than Norwegian, although the languages are closely related (both are part of the North Germanic language family), while languages as diverse as Chinese and Estonian displayed similar LIs. This raises the question whether these variations are due to methodological or sampling issues or indeed indicate valid differences in language laterality, which we attempt to answer in the following paragraphs.

The observed interaction of stimulus language and ear in the main sample (Analysis 1) suggests that “nativeness” of the stimulus language has an effect on the magnitude of the ear advantage, that is, performing the task with non-native (unfamiliar) as opposed to native (familiar) stimuli appears to produce a reduction in the measured laterality. The question however is whether the use of native vs. non-native stimuli can also explain the differences in the magnitude of the ear advantage observed between languages. Since the languages for which we had native syllables (English, German, Norwegian, Estonian) were selected arbitrarily, it might be that these selected languages (by chance) display stronger mean LIs than the other languages (specified as non-native). In this case, the main effect of “nativeness” would reflect true differences between languages in the LI rather than an effect of congruency between stimuli and native language. To differentiate between these opposing interpretations we further explored the data with regard to the phonetic overlap between the stimulus language and the native language of the participant. If the use of native vs. non-native stimuli alone was to explain the “nativeness”-effect, the following observations would be predicted: (1) in the languages for which native stimuli were used, there should be no effect of language background, and (2) in the languages for which non-native stimuli were used, a significant effect of phonetic overlap could be expected. However, neither of these predictions was right (see Analyses 2a/b; relevant effects not significant together with high test power), therefore the differences cannot be related to the stimulus-language congruency alone. Moreover, the largest language groups (English, Danish, Norwegian, Hindi, Chinese, Spanish) as well as the English dialect groups, showed variations of LIs (Analysis 3a/b), however, the latter emerged only as a trend. This suggests that the differences in the magnitude of the REA reflect true differences between the languages, whereas the differences between the English dialects are more subtle and therefore did not influence the REA substantially.

There are distinct linguistic features that may contribute to different degrees of lateralization. For example, there is evidence that speakers of tonal languages, such as Chinese, display greater variability in the degree and direction of language lateralization than non-tonal languages, such as English and Spanish (e.g., Valaki et al., 2004). In an fMRI study, Li et al. (2010) observed a rightward asymmetry in frontoparietal regions when native speakers of Mandarin Chinese were asked to make judgements about lexical tones relative to when subjects were making judgements about consonants and rhymes, indicating a more bilateral, less asymmetrical processing of language in tonal languages. This could be reflected in the present study, by the fact that native speakers of Chinese showed a smaller REA, compared to native speakers of English and Spanish. However, since Chinese is the only language that uses tone to distinguish between monosyllables in the present database, other explanations need to be considered. Languages also differ with regard to their rhythmic structure. Rhythm rests on a combination of different acoustic properties, but the parameter that is most often linked to rhythm in the literature is duration (Loukina, Kochanski, Rosner, Keane, & Shih, 2011). For example, Estonian and Finnish are languages that use vowel-duration changes to signal phonetic distinctions, and this function is largely carried out by the right hemisphere (Kirmse et al., 2008). This may be one explanation for the relatively small LI of the Estonian sub-sample. Finally, it is possible that the differences in LIs in the non-native stimulus sample were influenced by the level of English proficiency, with less proficient subjects being more left-lateralized in their second language (see Hull & Vaid, 2007). However, since we do not know the proficiency level of the participants, this hypothesis cannot be explored with the current data.

In addition, there were other factors (sex, handedness) that affected the magnitude of the REA, as expected based on previous studies (Hugdahl, 2003; Roup, Wiley, & Wilson, 2006; Voyer, 2011). In the main sample (Analysis 1), handedness appeared to affect language lateralization, with right-handers displaying stronger left-lateralization than left-handers, as being widely reported (see Corballis, 1989; Ocklenburg, Beste, Arning, Peterburs, & Güntürkün, 2014) and being in line with previous results obtained with the current paradigm (Hugdahl, 2003), or neuroimaging techniques (e.g., Knecht et al., 2000; Pujol, Deus, Losilla, & Capdevila, 1999; Westerhausen et al., 2006). Further, the sex effect, with males being more left-lateralized than females, was significant in the non-native stimulus sample (Analysis 2b) and is in accordance with previous dichotic listening studies (Bless et al., 2013; Hirnstein et al., 2013; Hiscock et al., 1994; for a review, see Voyer, 2011). It should be noted that both factors (sex and handedness) had only limited effects on the REA (as indicated by small effect sizes) and no interaction with language background, despite similar sample-composition (more than 60% males in 13 of 16 sub-samples, see Appendix Table A3). For example, Norwegian and Estonian samples had a balanced ratio of males and females, yet they displayed very different degrees of lateralization (18.3% and 6.1%, respectively); on the other hand, Hindi and Chinese had different sample compositions with regard to the factor sex, yet they showed similar degrees of lateralization (5.9% and 6.4%, respectively). There was no significant effect of age on the ear advantage, which is contrary to what might be expected based on previous reports (Bellis & Wilber, 2001; Gootjes, Van Strien, & Bouma, 2004; Roup et al., 2006).

In summary, the observed differences are stimulus-independent and related to the way the sounds are processed, as a more or less lateralized function, in native speakers of different languages. It may be that in some languages, e.g., tonal languages (Chinese) or languages with certain rhythmic characteristics (Estonian), a less lateralized processing of speech sounds is more efficient. In addition, laterality appears to be influenced by handedness and sex, although to a lesser degree than by language background; it may also vary within a person, e.g., as a function of hormonal fluctuations (e.g., Wadnerkar, Whiteside, & Cowell, 2008).

Beyond laterality, we also found that performing the task with non-native stimuli resulted in more errors (i.e., lower overall performance) than when native stimuli were used. Thus, analogously to what was discussed regarding the variability of the laterality, one might ask whether this is a methodological artefact of the “nativeness” of the stimulus material, or reflects “natural” variations across languages. For the former to be true, there should be: (1) no main effect of native language in the native stimulus sample, and (2) a main effect of phonetic overlap in the non-native stimulus sample, with a positive linear relationship between the overlap score and correct reports. Since the results showed a main effect of native language in both samples, but no systematic relationship between phonetic overlap and performance, it can be concluded that the different performance levels were not simply due to the “nativeness” of the stimulus material but also related to cross-linguistic variations. In addition, age had an effect on performance in all but the non-native stimulus samples. This is in line with previous reports that have shown a decline in cognitive performance with age (e.g., Passow et al., 2012; Salthouse, 2009).

### Limitations

Certainly, large-scale data collection using smartphones also has its limitations, mainly embodied in the lack of control over the experimental parameters. Some of the “noise”, however, can be removed by using strict exclusion criteria. For example, participants who only gave right-ear responses may have had a hearing loss or failed to plug in the left earbud and were excluded from the analysis.

Despite the global reach of the experiment, the current sample is not without bias towards English-speaking participants. However, this bias is significantly reduced to about 55%, compared to the 82% reported by Arnett (2008), and a further reduction is to be expected from future studies as smartphones become more widely available and affordable. Furthermore, although participants from 67 different native language backgrounds were part of the present study, many cultures were only represented in small numbers. For future experiments of this kind, it should be attempted to specifically target non-English, non-Western cultures, for example, by translating the app into various languages and deploying recruitment campaigns via social media and news platforms in those countries. Another bias is found in the fact that the current app only runs on iOS devices (e.g., iPhones). Although there is no evidence that suggests that Apple users would perform differently on a language-laterality test than e.g., Android users, such restriction should be avoided if possible, also considering the goal to reach a larger audience.

The present study does not answer beyond speculation as to why there are differences in language laterality between speakers of various native language backgrounds and English dialects (see discussion above). Thus, it may have been useful to include a few more items in the pre-test settings, for example, English proficiency level (see discussion above), or education level. However, since we intended this part to be short and simple in order to avoid overloading the participant with questions and information before the actual test started, we only included the most basic variables.

Finally, it may be argued that participants should only be tested with their native stimuli, to avoid a confounding effect of stimulus-language congruency. However, this study intended to collect a global sample, and as it was not possible to provide native syllables in all languages, some had to be tested with non-native stimuli. In addition, the present results showed that choice of stimulus language alone could not explain the observed differences in language lateralization between the language groups.

## CONCLUSIONS

This study reveals that the REA is a general perceptual effect, emerging across languages, but differing in magnitude between languages. These differences may be at least partly related to linguistic aspects of the languages themselves, with bias towards specific phonological features (e.g., vowel duration) in the native language or dialect affecting the processing of language as a more or less lateralized function. These results suggest that more emphasis should be placed on cultural and linguistic specificities of psychological phenomena, as suggested by Henrich et al. (2010), and on the need to collect more diverse, cross-cultural/cross-linguistic samples. The study further shows that smartphone-based data collection is an effective method to gain access to larger and more diverse populations.

## APPENDIX

**TABLE A1:** Analyses overview

	Research question	Sample	*N*	Exclusion criteria	ANOVA-statistics
Analysis 1	Is the REA universal?	Main sample	4408	General^a^	Dependent variable: right ear/left ear scoresFactors: sex, handedness, stimulus languagecovariate: age
Analysis 2a	Is the size of the REA driven by the “nativeness” of the stimulus language?	Native-stimulus sample (100% phonetic overlap)	2348	General^a^Non-right-handers	Dependent variable: right ear/left ear scoresFactors: sex, native language Covariate: age
Analysis 2b		Non-native stimulus sample (50%-100% phonetic overlap)	942	General^a^Native languages with *N* < 30Non-English stimuliNon-right-handers	Dependent variable: right ear/left ear scoresFactors: sex, phonetic overlap Covariate: age
Analysis 3a	Does the REA vary across languages/dialects?	Main sample	3028	General^a^Native languages with *N* < 100Non-right-handers	Dependent variable: right ear/left ear scoresFactors: sex, native language Covariate: age
Analysis 3b		Native-English sample	2022	General^a^Native languages with *N* < 30Non-right handers	Dependent variable: right ear/left ear scoresFactors: sex, dialect Covariate: age

^a^See Database and samples section for list of general exclusion criteria.

*N* = number of subjects, REA = Right-ear advantage.

**TABLE A2:** Phonetic overlap between native language and stimulus language (English)

Language	Bilabial		Alveolar		Velar		Phonetic overlap
English (stimulus)	p	b	t	d	k	g	−
Afrikaans	p	b	t	d	k	g	6
Chinese	p	−	t	−	k	−	3
Danish	p	−	t	−	k	−	3
Dutch	p	b	t	d	k	−	5
French	p	b	t	d	k	g	6
Hindi	p	b	t	d	k	g	6
Italian	p	b	t	d	k	g	6
Russian	p	b	t	d	k	g	6
Spanish	p	−	t	−	k	g	4
Swedish	p	b	t	d	k	g	6

Mixed sample (*N* > 30).

**TABLE A3:** Laterality indices by native language

Native language		*N*	LI (mean ± SE)	Errors (mean ± SE)	Composition (sex, mean age)
ENGLISH	North American British Australian ALL	1526 419 77 2049	15.8 (± 0.7) 12.5 (±1.3) 17.1 (± 3.3) 15.2 (± 0.6)	7.5 (± 0.1) 8.5 (± 0.2) 7.3 (± 0.5) 7.7 (± 0.1)	65% M, 36 y 70% M, 32 y 75% M, 35 y 66% M, 35 y
Danish		333	11.6 (± 1.5)	8.0 (± 0.2)	68% M, 29 y
Norwegian		252	18.3 (± 1.7)	4.9 (± 0.2)	55% M, 34 y
Hindi		141	5.9 (± 2.4)	11.4 (± 0.3)	84% M, 30 y
Spanish		127	10.3 (± 2.0)	11.9 (± 0.4)	74% M, 34 y
Chinese		145	6.4 (± 2.4)	8.8 (± 0.4)	37% M, 26 y
Estonian		76	6.1 (± 2.0)	8.0 (± 0.5)	50% M, 28 y
Swedish		69	12.9 (± 2.9)	7.7 (± 0.5)	61% M, 35 y
Afrikaans		58	17.8 (± 3.3)	8.2 (± 0.5)	72% M, 34 y
German		44	15.5 (± 3.5)	7.6 (± 0.4)	80% M, 37 y
Russian		42	11.1 (± 3.4)	10.7 (± 0.7)	64% M, 27 y
French		35	9.7 (± 4.4)	10.2 (± 0.8)	71% M, 38 y
Dutch		35	13.5 (± 3.7)	7.2 (± 0.5)	77% M, 37 y
Italian		31	4.5 (± 5.4)	11.1 (± 0.9)	77% M, 40 y

Main sample (*N* > 30; only right-handers). *N* = number of subjects, SE = standard error, M = males, y = years, LI = laterality index.
